# Comparing Sediment Microbiomes in Contaminated and Pristine Wetlands along the Coast of Yucatan

**DOI:** 10.3390/microorganisms9040877

**Published:** 2021-04-20

**Authors:** Herón Navarrete-Euan, Zuemy Rodríguez-Escamilla, Ernesto Pérez-Rueda, Karla Escalante-Herrera, Mario Alberto Martínez-Núñez

**Affiliations:** 1 UMDI-Sisal, Facultad de Ciencias, Universidad Nacional Autónoma de México, Parque Científico y Tecnológico de Yucatán, Sierra Papacal-Chuburna Km 5, Mérida, Yucatán 97302, Mexico; heronnavarrete@hotmail.com (H.N.-E.); zuemy.rodriguez@gmail.com (Z.R.-E.); susana_escalanteh@hotmail.com (K.E.-H.); 2 Instituto de Investigaciones en Matemáticas Aplicadas y en Sistemas, UNAM, Unidad Académica Yucatán, Mérida, Yucatán 97302, Mexico; ernesto.perez@iimas.unam.mx

**Keywords:** coastal prokaryotic communities, metagenomic, Yucatan Peninsula

## Abstract

Microbial communities are important players in coastal sediments for the functioning of the ecosystem and the regulation of biogeochemical cycles. They also have great potential as indicators of environmental perturbations. To assess how microbial communities can change their composition and abundance along coastal areas, we analyzed the composition of the microbiome of four locations of the Yucatan Peninsula using 16S rRNA gene amplicon sequencing. To this end, sediment from two conserved (El Palmar and Bocas de Dzilam) and two contaminated locations (Sisal and Progreso) from the coast northwest of the Yucatan Peninsula in three different years, 2017, 2018 and 2019, were sampled and sequenced. Microbial communities were found to be significantly different between the locations. The most noticeable difference was the greater relative abundance of Planctomycetes present at the conserved locations, versus FBP group found with greater abundance in contaminated locations. In addition to the difference in taxonomic groups composition, there is a variation in evenness, which results in the samples of Bocas de Dzilam and Progreso being grouped separately from those obtained in El Palmar and Sisal. We also carry out the functional prediction of the metabolic capacities of the microbial communities analyzed, identifying differences in their functional profiles. Our results indicate that landscape of the coastal microbiome of Yucatan sediment shows changes along the coastline, reflecting the constant dynamics of coastal environments and their impact on microbial diversity.

## 1. Introduction

Coastal and estuarine ecosystems are among highly productive and diverse on Earth, where prokaryotes are the most abundant organisms [1,2]. Within coastal and estuarine ecosystems there are processes that mediate the transfer of carbon from land to sea, releasing a considerable amount of CO_2_ to the atmosphere, and sequestering carbon in sediments [3]. It has been reported that biomass and taxon richness of microbes are much higher in coastal sediments than in the corresponding water bodies [4]. Microbes play key roles in coastal marine ecosystem productivity, as well as in the regulation of relevant processes in the global biogeochemical cycles, and through anaerobic respiratory processes contribute to nitrogen, sulfur, iron, and carbon transformations [3]. The sediments provide complex nutrients for microbial growth, by deposition of organic matter from the upper water layer. In turn, rhizospheric and sediment microbes are the major biological components that contribute to the productivity of coastal ecosystems because degrade organic matter and release elemental nutrients, such as nitrogen and phosphorus, facilitating the growth of primary producers, i.e., algae [4,5]. Marine microbes are extremely sensitive to environmental changes and are generally the first responders to environmental perturbation because of their fast growth rates and genome plasticity [1]. Thus, the study of microbial community diversity, and their fluctuations over spatial and temporal scales, represents a useful tool to evaluate marine ecosystems [6].

Yucatan is a Mexican state located in the SE portion of the Gulf of Mexico, on Yucatan Peninsula, which is a limestone platform with a surface area of about 165,000 km^2^ and includes 365 km of coast with a strip that reaches up to 20 km inland from the coastline, where converges the Caribbean Sea and Gulf of Mexico. There are present diverse ecosystems that run parallel to the coast, starting with a coastal plain, followed by a barrier island with beaches and dunes. Inside the sandy barrier there are lagoons and wetlands [7]. The coastal territory represents 15% of the surface of the Yucatan state, living in it 6.5% of the population. Sixty percent of the coastal strip is under protection through two Biosphere Reserves, Celestún and Río Lagartos, as well as two state Protected Natural Areas, El Palmar and Bocas of Dzilam de Bravo. The necessity for information on the coastal ecosystem of Yucatan Peninsula is especially important when ecotourism and fisheries, among others economically activities occur in the zone, exerting a constant pressure on the biological diversity of the region. Therefore, it is interesting to compare the communities of microorganisms that live on the Yucatan coast, in sites with anthropogenic pressure and others that do not. This study describes the composition and abundance of species present in the microbial communities of Yucatan coast, as well as their functional profiles through 16S rRNA gene amplicon sequencing.

## 2. Materials and Methods

### 2.1. Site Description and Sample Processing

To carry out the description of the microbial communities present in the sediments of four wetlands of the Yucatan state, the wetland of Sisal (21°09′43.6″ N 90°02′27.2″ W) and Progreso (21°16′37.6″ N 89°40′35.6″ W) locations were selected because they are contaminated with organic and inorganic garbage, septic tank sedimentation sludge, sanitary waste, insecticides, and petroleum hydrocarbons (Supplementary Figure S1). Indeed, the concentration of total petroleum hydrocarbons (TPH) in Progreso and Sisal is exceeding the permissible level in coastal sediments of 70 μg/g [8]. By other side, two locations with not perturbations were sampled. These sites are located at the ecological reserve of El Palmar (21°08′56.4″ N 90°06′07.0″ W) and Bocas de Dzilam (21°27′22.2″ N 88°40′53.7″ W). Three sediment samples were taken for each location, for which three spots were chosen within a box meter square: one spot at the center and two more at the ends. The samples were extracted approximately 20 cm deep, and 2 g of sediment were taken from each spot and mixed with 6 mL of the LifeGuard Soil Preservation buffer (Qiagen Inc., Hilde, Germany) and stored at −20 °C. The experiment was conducted at three different times, between May 2017 and March 2018, in Sisal and Palmar locations; and October 2019 in Progreso and Bocas de Dzilam locations.

### 2.2. DNA Extraction and Sequencing

For DNA extraction it was used Qiagen MagAttract PowerSoil DNA KF Kit (Qiagen Inc., Hilde, Germany) following the manufacturer’s recommendations. After quality and purity evaluation of extracted DNA, the V3–V4 region of the 16S ribosomal RNA (rRNA) gene was amplified using the bacteria-specific primer pair 357wF (5′-CCTACGGGNGGCWGCAG-3′) and 785R (5′-GACTACHVGGGTATCTAATCC-3′). The primers used in the present study have an amplification bias for bacterial organisms. However, their design contains degenerate positions that allow non-bacterial sequences to be amplified, such as archaeal sequences, although to a lesser extent than bacterial ones. Therefore, archaeal gene fragments can be amplified and sequenced together with Bacteria in one experiment, such as it has been previously reported [9,10]. Amplifications were performed in 25 μL reactions with Qiagen HotStar Taq master mix (Qiagen Inc., Valencia, CA, USA), 1 μL of each 5 μM primer, and 1 μL of template, reactions were performed on ABI Veriti thermocycler (Applied Biosytems, Carlsbad, CA, USA). Amplification products were visualized with eGels (Life Technologies, Grand Island, NY, USA). Products were then pooled equimolar, and each pool was size selected in two rounds using SPRIselect Reagent (Beckman Coulter Inc., Indianapolis, IN, USA) in a 0.75 ratio for both rounds. Size selected pools were then quantified using the Qubit 4 Fluorometer (Life Technologies, Grand Island, NY, USA) and loaded on an Illumina MiSeq (Illumina Inc., San Diego, CA, USA) 2 × 300 flow cell at 10 pM. DNA extraction and metagenomic sequencing were requested from the Research and Testing Laboratory (Lubbock, TX, USA).

### 2.3. Data Analysis

Of the six samples taken in 2017, three at El Palmar and three at Sisal, only two were analyzed, one from each location, and four were discarded for having a low number of reads. Of the six samples taken in 2018, three at El Palmar and three at Sisal, only four were analyzed, two from each location. Two samples that did not have enough reads were discarded, one from each location. While the six samples taken in 2019, three at Bocas de Dzilam and three at Progreso, all were analyzed. Therefore, we analyzed 12 samples from four different locations: two samples from 2017 (1 from El Palmar, 1 from Sisal), four samples from 2018 (2 from El Palmar, 2 from Sisal), and six samples from 2019 (3 from Bocas de Dzilam, 3 from Progreso) (Supplementary Table S1). Data processing was performed using Quantitative Insights into Microbial Ecology 2 v2021.2 (QIIME 2) pipeline [11]. First, paired end sequences were imported into QIIME2. Then Divisive Amplicon Denoising Algorithm 2 (DADA2) [12] was used with the reads to perform PhiX sequence filtering, chimera sequence elimination, pairing, and Amplicon Sequence Variants (ASVs) detection. The forward sequences were truncated to 285 base pairs because the quality of reads after base 285 declined, and to reverse sequences were truncated to 201 base pairs for the same reason. Subsequently, the taxonomic classification of ASVs was performed using a Naive Bayes fitted classifier through the q2-feature-classifier plugin. The classifier was trained with the SILVA v132 99% 16S sequence database [13] and the 357wF/785R set of primers used in this study. The obtained ASVs were taxonomically annotated using the "classify-sklearn" command of q2-feature-classifier plugin, with the default confidence level of 0.7. A phylogenetic tree was inferred by using the QIIME 2 align-to-tree-mafft-fasttree plugin. We obtained a multiple sequence alignment of ASVs sequences using Multiple sequence Alignment based on Fast Fourier Transform (MAFFT) [14], and the phylogenetic tree was inferred by applying the maximum-likelihood procedure implemented in Fasttree 2 [15]. In Supplementary Figure S2 the variation of the relative abundance values can be observed.

The ASVs table was normalized by using rarefaction for diversity analysis. A series of alpha diversity indices were calculated on rarefied ASVs for all samples using the R package phyloseq [16], such as rarefaction curves, Chao estimator, and Shannon index. The Double Principle Coordinate Analysis (DPCoA) [17] was used to measure beta diversity in samples. Functional profiles of microbial communities were predicted by Phylogenetic Investigation of Communities by Reconstruction of Unobserved States version 2 (PICRUSt2) [18,19] from observed data of the taxa identified using the 16S rRNA reads analyzed with QIIME 2. Functional predictions were assigned up to all Kyoto Encyclopedia of Genes and Genomes (KEGG) [20] orthology (KO) numbers, to obtained KEGG pathway abundance information. The taxonomic groups identified, as well as the predicted metabolic functions for the microbial communities were statistically analyzed to assess the existence of significant differences in their relative proportions. To carry out the statistical evaluation we used the Statistical Analysis of Metagenomic Profiles (STAMP) software 2.1.3 [21]. A White’s non-parametric t-test was implemented for hypothesis testing. Results with *p*-value < 0.05 were considered as significant for both taxonomic and metabolic data.

## 3. Results

### 3.1. Taxonomic Composition of Prokaryotic Communities of the Yucatan Coast

The taxonomic community composition was determined from the amplicon sequencing of 16S rRNA gene fragments using the QIIME 2 software [11] and the Silva RNA database [13]. At the domain level, sequence reads were mostly assigned to Bacteria domain, which was numerically dominant in the four sites, with a minimum proportion of 86.61% for Bocas de Dzilam and a maximum of 99.91% for Sisal sample of 2018. By contrast, the representation of Archaea domain was maximum for the sample taken in Bocas de Dzilam (13.38%), and a minimum on Sisal (0.09%). At 99% similarity, phylogenetic assignments resulted in the identification of 49 phyla, of which 40 phyla have a known cultivable representative, such as Acidobacteria, Chloroflexi, Firmicutes, and Proteobacteria; whereas 9 phyla do not have cultivable representative, so far, such as CK-2C2-2, BRC1, WOR-1, and WS2. In addition, 99 classes, 177 orders, 178 families, and 179 genera were identified (Supplementary Table S2).

**Figure 1:**
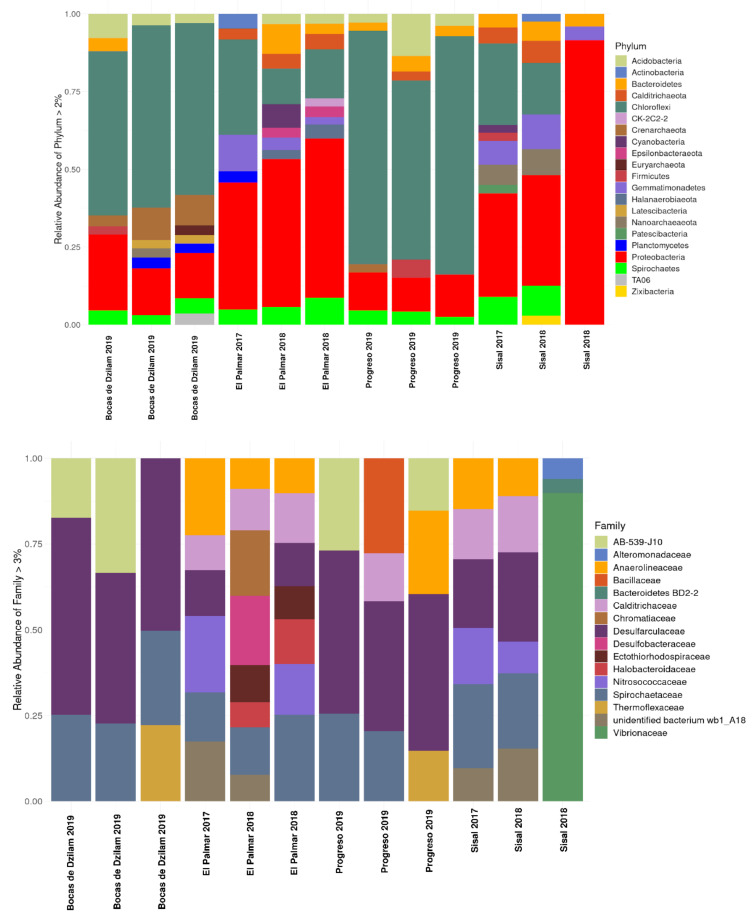
Relative abundance of bacteria composition across the four wetland sampling sites at phylum (**upper**) and family (**bottom**) levels. Conserved sites: Bocas de Dzilam and El Palmar; contaminated sites: Progreso and Sisal. X-axis: sampled locations (including the year). Y-axis: relative abundance of taxon.

The relative abundances of microbial taxa at the phylum level showed that about 80% of bacterial sequences were assigned to Proteobacteria, Chloroflexi, Gemmatimonadetes, Spirochaetes, Bacteroidetes, Actinobacteria, Calditrichaeota, and Acidobacteria (Figure 1, upper panel). The phylum Chloroflexi presents a higher abundance in the samples taken in 2019 from Bocas de Dzilam and Progreso, while Proteobacteria present their highest abundance at El Palmar and Sisal (samples 2017 and 2018). The differences observed between the phyla when comparing the samples are evident with respect to the years of sampling rather than with respect to the studied sites. The phyla Archaea identified in the samples were Crenarchaeota, Nanoarchaeota, Euryarchaeota, Asgardaeota, and Diapherotrites (Supplementary Table S2). Among these, those that had a relative abundance greater than 2% were Crenarchaeota and Euryarchaeota, present in Bocas de Dzilam and Progreso; and Nanoarchaeota present in Bocas de Dzilam and Sisal. At the family level, the most abundant in the four sites and with cultivable organisms were Anaerolineaceae, Bacteroidetes, Calditrichaceae, Chromatiaceae, Desulfarculaceae, Desulfabacteraceae, Halobacteroidaceae, Nitrosococcaceae, Spirochaetaceae, and Vibrionaceae (Figure 1 bottom pannel). Of the previous families, Vibrionaceae exhibits the highest abundance in Sisal sample of 2018, with 79.25%, while in the other three sites the abundance of this family was zero. While Desulfarculaceae had a high abundance in the samples obtained in 2019 from Bocas de Dzilam and Progreso. Organisms belonging to families with not cultured representatives, such as AB-539-J10 were identified in Bocas de Dzilam and Progreso; and wb1 A18 in Sisal and El Palmar.

### 3.2. Diversity Estimates

**Figure 2:**
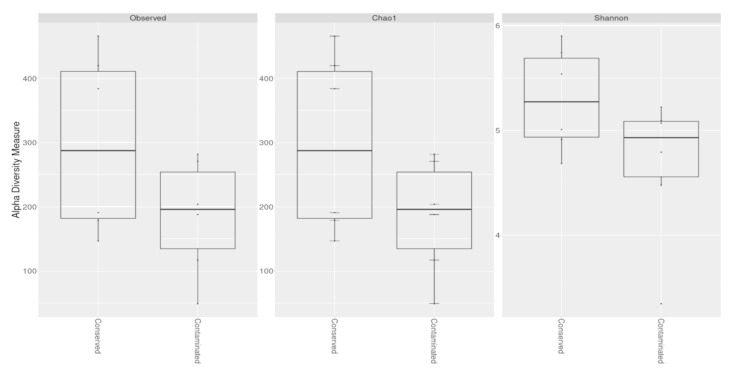
Alpha diversity measures of the microbial communities of conserved sites (Bocas de Dzilam and El Palmar) and contaminated sites (Progreso and Sisal). **Left:** Observed diversity; **Center:** Chao 1 estimator diversity, the bar shows the standard error of the estimator; **Right:** Shannon index diversity. X-axis: Analyzed environments. Y-axis: Alpha diversity measure.

To evaluate the diversity present in the locations, the alpha diversity in the samples was estimated. According to the Chao 1 estimator, the diversity analysis of the samples from the conserved locations (Bocas de Dzilam and El Palmar) shows a highest value than those sites impacted by anthropogenic activities (Progreso and Sisal). The diversity in all the samples from the conserved sites shows a Shannon Indexes between 4.6 and 5.8; indicating a balanced distribution of diversity. In contrast, the samples from contaminated sites show a decrease in diversity, with values between 3.3 and 5.2 (Figure 2; Supplementary Table S3). Despite a decrease in the estimation of the diversity of contaminated sites, there is an overlap of the highest values of the contaminated areas with the lowest values of the conserved areas. In this regard, sites contaminated with sewage, construction materials, plastic debris and human garbage present a high diversity of microorganisms [22]. Thus, contamination can explain the high values in the Shannon index of diversity of contaminated sites like Progreso. A rarefaction analysis was carried out based on phylogenetic annotation of reads and using vegan function of phyloseq package (Supplementary Figure S3). Rarefaction curves obtained at species level showed that all samples reach a plateau, which indicates that the sequencing depth was sufficient to carry out a thorough description of each sample, as well as the description of a good proportion of the microbial diversity in these wetlands soils.

**Figure 3:**
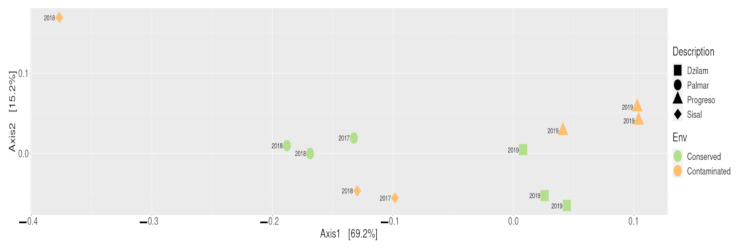
DPCoA of contaminated (Progreso and Sisal) and conserved (Dzilam and El Palmar) locations. Scatterplot of the first two principal axes of the DPCoA. Each geometric figure represents an individual sample, square: Bocas de Dzilam; circle: El Palmar; triangle: Progreso; diamond: Sisal. Each color represents an environmental state; green: conserved; orange: contaminated.

We next attempted to determine how similar are the samples obtained from the different sites in terms of their identified microbial communities. To this end, we used Double Principal Coordinates Analysis (DPCoA) [17] to examine the differences among species using a dissimilarity matrix, and the species distribution among communities using an abundance/absence matrix (Figure 3). DPCoA shows the effect of the sampling year, which seems to exert a greater influence on the grouping of microbial communities than the environmental state or the sampling site. It can be seen that the samples from 2018 to 2017 are clustered to the left of the samples of 2019, on axis 1. While the effect of the environmental states is arranged approximately in order along the second axis. Changes in physico-chemical variables such as temperature, salinity or ocean currents that occur throughout the year and between different years seem to have a greater impact on the conformation of the structure of microbial communities, than variations caused by anthropogenic impacts.

**Figure 4:**
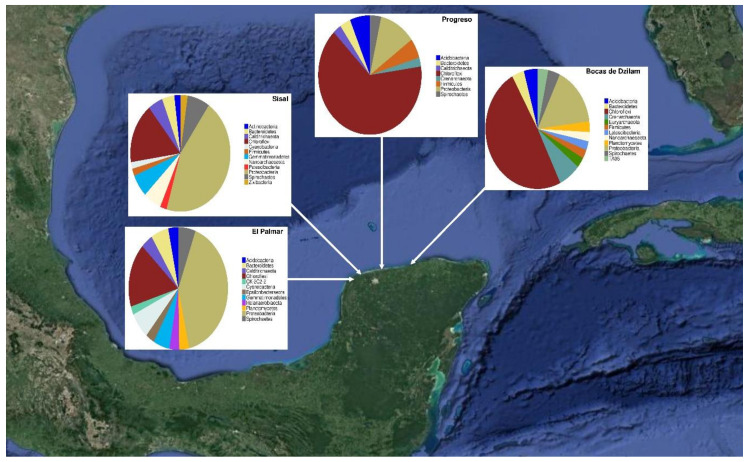
Microbial landscape of the Yucatan coast. Changes in the diversity and abundance of microbial species present in four locations on the Yucatan coast are observed.

The differentiation of the prokaryotic communities is not only in the number of phylotypes identified, but also in their relative abundance. For instance, the phylum Chloroflexi was the most abundant in the samples from Bocas de Dzilam and Progreso, follow by the Proteobacteria. While in the samples from El Palmar and Sisal is the opposite case, the phylum Proteobacteria was the most abundant, and then the phylum Chloroflexi (Figure 4). These data lead us to observe a change in the microbial communities of the Yucatan coast, indicating heterogeneous microbial landscapes.

### 3.3. Environment–Taxonomy Association

**Figure 5:**
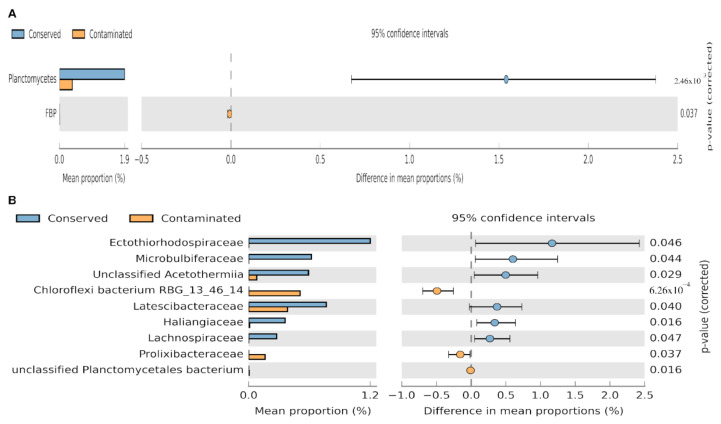
Metataxonomic profile comparisons of differentially phylotypes between conserved sites (Bocas de Dzilam and El Palmar) and contaminated sites (Progreso and Sisal) using Statistical Analysis of Metagenomic Profiles (STAMP) analysis. (**A**): Phylum level. (**B**): Family level. A positive difference between proportions denotes a greater abundance in conserved group (blue bar), whereas a negative difference between proportions shows a greater abundance in contaminated group (orange bar) for the given phyla or family; the standard deviation is shown as a bar in the circles.

In order to determine if there is any association between the taxonomic groups identified in our data and the environment from which they were sampled, a statistical analysis was carried out using the Statistical Analysis of Metagenomic Profiles (STAMP) [21] software. This analysis was performed using the taxonomic levels of phylum and family, for which we pooled samples and compared relative abundances between conserved (*n* = 6) and contaminated (*n* = 6) sites. From this analysis, we found that Planctomycetes phylum was overrepresented in the conserved locations, while the candidate phylum FBP was the one with an enrichment in its abundance in the contaminated locations (Figure 5A).

For the conserved sites, similar trends have been described, such as the Mai Po Ramsar wetland in Hong Kong and the National Nature Reserve of the Beilun estuary in Guangxi, China, where the sediments are well-preserved and for which a high abundance of Planctomycetes has been reported [23,24,25]. Planctomycetes are sensitive to chlorinated organic pollutants, such as pentachlorophenol used as a pesticide, causing a decrease in their abundance [26] in sites contaminated by anthropogenic activities. Therefore, the presence of Planctomycetes can be an indicator of the absence of contaminants. The candidate phylum FBP is widespread in extreme environments on Earth, including wastewater sites. Phenotypic and genome analysis indicated an extreme resistance against toxic compounds, which enables members of this phyla to be able to inhabit a variety of contaminated environments on Earth [27,28]. Analysis at the family level, reveals a large proportion of Ectothiorhodospiraceae, Microbulbiferaceae, unclassified Acetothermiia, Latescibacteraceae, Haliangiaceae, and Lachnospiraceae, in the conserved locations. While in contaminated locations, the three families with the highest abundance were Chloroflexi bacterium, Prolixibacteraceae, and unclassified Planctomycetales. The families found in both environments have been reported as present in marine or coastal environments; except Lachnospiraceae family, which is part of human gut microbiota [29]. In the case of Acetothermiia, Latescibacteraceae, or Haliangiaceae are novel families identified in metagenomics analysis [30,31,32,33,34,35]. Finally, families found in our contaminated sites, were previously reported in other contaminated environments [36,37,38,39,40].

### 3.4. Functional Profile Prediction

**Figure 6:**
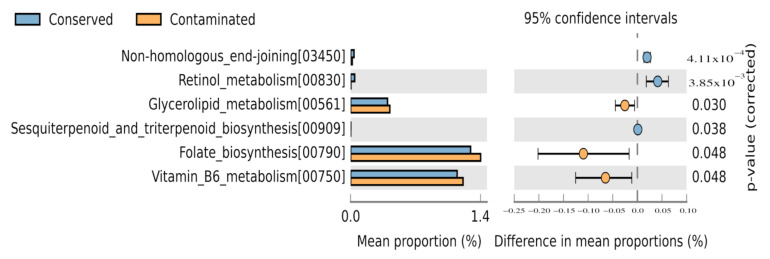
Metabolic pathways profile comparisons between conserved sites (Bocas de Dzilam and El Palmar) and contaminated sites (Progreso and Sisal) using STAMP analysis. Analyses were carried out at level 3 of specific pathway associated with a specific function using KEGG. Blue bar: conserved sites; orange bar: contaminated sites. The standard deviation is shown as a bar in the circles.

To evaluate the possible molecular functions of the prokaryotic communities identified in the present work, we achieved a prediction of their metabolic capabilities. The PICRUSt2 software was used to predict the metabolic capabilities of the identified microorganisms, together with the KEGG database to carry out functional annotation at level 3: specific pathway associated with a specific function. To identify the statistically significant pathways, we carried out an analysis using STAMP and setting as significant those molecular functions with a *p*-value < 0.05. For which, we pooled the samples and compared relative abundances between conserved (*n* = 6) and contaminated (*n* = 6) sites, as was done for the analysis of the environment–taxonomy association (Figure 6).

We observed that in conserved locations the metabolic pathways non-homologous end joining, retinol metabolism, and sesquiterpenoid and triterpenoid biosynthesis were significantly represented. Non-homologous end joining (NHEJ) is a mechanism to rectify DNA double-strand breaks which are one of the most lethal forms of DNA damage. NHEJ repair pathway was initially identified in mammalian cells and was considered to be restricted to eukaryotes, but today there is evidence for the presence of NHEJ system in a variety of phylogenetically diverse prokaryotic genomes [41,42,43]. Retinol is part of retinoids, which are lipophilic isoprenoids composed of a cyclic group and a polyunsaturated side chain, which contain an alcohol functional group. The synthesis of retinoid intermediates such as retinol has been reported by different researchers in archaeal organisms such as *Halobacterium halobium*, *Haloarcula marismortui*, *Haloquadratum walsbyi*; as well as in the bacteria *Salinibacter ruber*, *Bacillus cereus*, and uncultured marine bacterium HF10 49E08. In particular, the halophilic bacterium *S*. *ruber* has a retinal-based light-driven pump known as xanthorhodopsin [44,45,46]. Sesquiterpenoid and triterpenoid have commercial potential in the pharmaceutical sector due to their bioactivities such as antiviral, anticancer, anti-inflammatory, wound-healing properties, pigments, hormones and signaling molecules, antibiotics, antifeedants, or pollinator attractants. Terpene synthases (TPSs) genes are widely distributed in bacteria [47]; while Sesquiterpene Synthases genes have been described in *Nostoc* sp. strain PCC 7120, *Nostoc punctiforme* PCC 73102 and *Streptomyces* [48,49]. In contaminated sites, glycerolipid metabolism, folate biosynthesis, and vitamin B6 metabolism were significantly represented. The glycerolipid metabolism is important in bacteria because it play key structural and functional roles in the bacterial membrane. Major constituents of the bacterial cell wall such as phosphatidylethanolamine (PE), phosphatidylglycerol (PG), and cardiolipin (CDL) as well as precursor for fatty acid biosynthesis 1,2 diacyl-sn-glycerol are produced through this pathway [50,51]. Modifications of the bacterial membrane have been reported as a mechanism to deal with contamination of the environment inhabited by bacteria, since it is a first site of contact between the cell and toxic compounds [52]. Therefore, the glycerolipid metabolism is an important metabolic route of environmental adaptation of bacterial communities that inhabit contaminated sites, such as those analyzed here. Folates, present in many pathogenic microorganisms [53], such as those found in contaminated sites, must be synthesized de novo through the folate biosynthetic pathway. Furthermore, pathogenic microorganisms such as *Mycobacterium tuberculosis* synthesize pyridoxal 5-phosphate (PLP), the bioactive form of vitamin B6, which is essential for the growth and survival of the pathogen [54].

### 3.5. Environmental Metabolic Pathways

**Figure 7:**
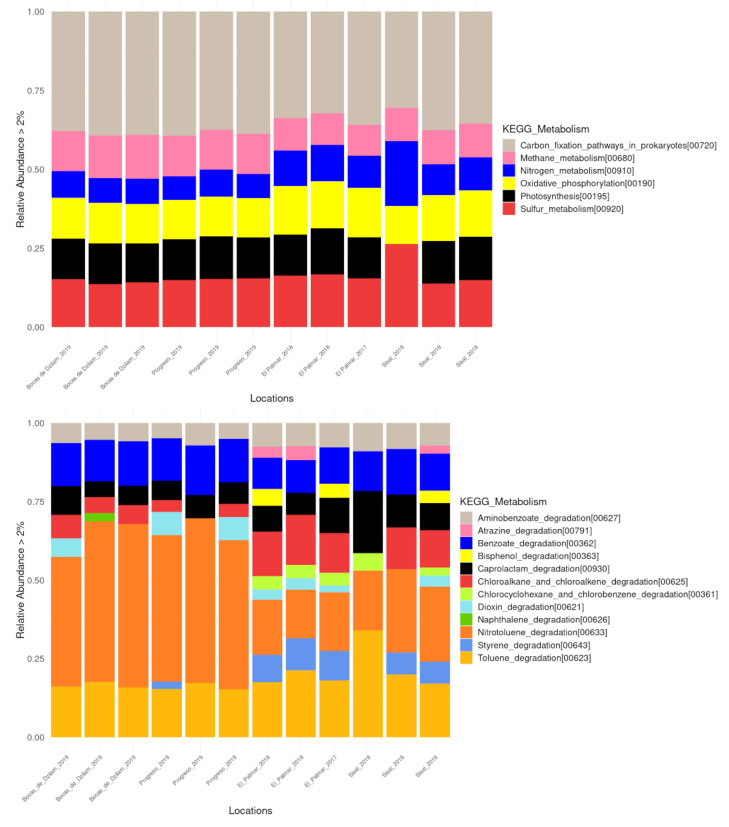
Metabolic pathways associated to biogeochemical (upper) and xenobiotics (bottom) functions.

Prokaryotes in wetland sediments play crucial roles in biogeochemical processes including carbon, nitrogen, and sulfur cycling. The oxidant ammonium bacteria, methane and sulfide use the O2 limited in the oxic/anoxic interface of wetlands sediments, which influences other biogeochemical cycles of wetlands [55]. In all the localities analyzed here, methane, nitrogen, and sulfur metabolism were present; as well as the metabolic functions of carbon fixation, photosynthesis, and oxidative phosphorylation (Figure 7, upper panel; Supplementary Figure S5). Within the nitrogen (N) cycle, oxidation of ammonium (NH4+) or ammonia (NH3) to nitrite and its subsequent conversion to nitrate is a primary activity [56]. Several bacteria have been reported as ammonia oxidants, most of which belong to phylum Proteobacteria. It was believed that this process was only controlled by ammonia oxidizing bacteria (AOB), but the discovery of ammonia oxidizing archae (AOA) has modified this notion [57,58]. About 69% of all methane sources is the result of microbial metabolism, and its production typically occurs in sites where organic matter is decomposed in the absence or limitation of oxygen. Wetlands are the single largest source of microbial methane, and the amount of methane is the result of the balance between production (methanogenesis) and their oxidation (methanotrophy) [59,60]. These processes are carried out mainly by organisms of different cellular domains, Archaea and Bacteria, respectively. The sulfur cycle consists of an oxidative and a reductive stage. This cycle is performed by sulfate reducing and oxidizing bacteria. On the reductive step, sulfate functions as an electron acceptor in metabolic pathways and is converted to sulfide. On the oxidative step, reduced sulfur compounds such as sulfide serve as electron donors for phototrophic or chemolythotrophic bacteria to convert these compounds to elemental sulfur or sulfate [61]. In coastal environments, methanogens are outcompeted by sulfate-reducing bacteria (SRB) for acetate-type precursors and hydrogen [62]. The relative abundance of methane and sulfur metabolism is similar in all the locations, as can be seen in Figure 7 (upper panel). In general, the metabolic processes associated with the biogeochemical cycles that we have found in our analysis show a similar relative abundance in all the sampled locations.

As part of the results of the functional profiles, we identified metabolic pathways associated with the degradation of xenobiotics (Figure 7, bottom panel; Supplementary Table S4). Three-quarters of the identified pathways are associated with the degradation of aromatic hydrocarbons, such as aminobenzoate, benzoate, naphthalene, nitrotoluene, and toluene; or any of its derivatives such as bisphenol, caprolactam and styrene. Aromatic compounds comprise one-quarter of the earth’s biomass and are the second most widely distributed class of organic compounds in nature next to carbohydrates [63,64,65]. Nitrotoluene and toluene degradation was present in all locations, but to a greater extent in samples obtained from Bocas de Dzilam and Progreso. While aromatic derivatives such as styrene and bisphenol, were identified only in the samples from Palmar and Sisal locations. Other metabolic pathways associated to xenobiotic degradation were chloroalkane and chloroalkene degradation, chlorocyclohexane, and chlorobenzene degradation. These chlorinated hydrocarbons are abundant in sedimentary environments and can have origins such as phytoplankton, marsh vegetation; or come from industrial metallurgical and textile pollutants, being anthropogenic organic compounds widely used in synthetic processes in industry [66,67].

## 4. Discussion

Wetlands of Yucatan coast are ecologically important ecosystems that are under severe pressure because of environmental changes and anthropogenic activities. Earlier studies using metagenomic approaches have revealed that human activities affect the microbial communities present in the mangrove sediment that are crucial for the health and balance of this ecosystem [22,68,69]. Our study describes for the first time the structure of prokaryotic communities from anthropogenically influenced coastal wetland ecosystems and conserved wetland ecosystems in Yucatan Peninsula. For the wetlands of Sisal and Progreso, and those of the Ecological Reserve of El Palmar and Bocas de Dzilam, the hypothesis of having a different distribution of microbial species was proposed, due to the anthropogenic activities that affect differently to microbial communities present in the sediments of mangroves of the Yucatan coast. We found a different composition in some prokaryotic taxa, which are related to the places where they live. For instance, in samples obtained from contaminated wetland of Progreso we found the phylum Chloroflexi and Actinobacteria (Supplementary Figure S4) with a statistically significant abundance when compared with the conserved wetland of Bocas de Dzilam. When we pooled samples from contaminated sites and compared them with conserved sites, the candidate phylum FBP was found with significant abundance in contaminated wetland. In the case of the Ecological Reserve El Palmar, the abundance of phylum Acidobacteria was statistically significant in comparison to Sisal. When we compared Bocas de Dzilam and Progreso samples, the phylum statistically significant were Crenarchaeota, Planctomycetes, Nanoarchaeota, Zixibacteria, Acetothermia, Patescibacteria, and Asgardaeota in the conserved locations (Supplementary Figure S4). In an overview way, when we pooled samples from conserved sites to compare against contaminated sites, the phylum with the significant abundance was Planctomycetes. This last result agrees with similar studies carried out in preserved mangroves [23,24,70]. In the case of the Archaea domain, the highest abundance found in our study was 8.3%, corresponding to the Crenarchaeota phylum. However, this abundance may be underestimated, since the primers used in our analysis preferentially amplify bacteria, and to a lesser extent archaeal sequences. The abundance and diversity of the Archaea domain present in coastal environments have been widely reported [55,71,72,73,74,75]. Therefore, analyzes focused on the identification of organisms belonging to this domain are necessary, which will allow a more complete panorama of the prokaryotic communities that are part of the microbiome landscape of the Yucatan coast.

The results of beta diversity analysis showed that prokaryotic communities present in the wetlands of Bocas de Dzilam and Progreso are more similar to each other, despite having different environmental states. Similarly, the microbial communities present in Sisal and El Palmar showed greater similarity between them. Thus, it seems that the temporal variable, that is, the sampling year, seems to be a very important factor in the formation of the structure of microbial communities. As observed in the DPCoA, in which the samples from 2017 to 2018 were clustered and separated from those obtained in 2019. The changes that occur between the different years in physicochemical variables such as salinity, temperature or ocean currents seem to have a greater weight in determining the clades present in the sampled sites, as well as in their proportion. While the environmental states of conservation and anthropogenic contamination showed to have less influence on the structure of the microbial communities that inhabit the studied sites.

To determine the possible molecular functions of the prokaryotic communities, we made a prediction of their metabolic capabilities and their statistical evaluation to analyze the possible environmental associations. With respect to pathways identified in contaminated wetlands, glycerolipid metabolism, folate biosynthesis, and vitamin B6 metabolism were significantly represented. These molecular functions play a key role in the mechanism of adaptation of bacteria to stressful environments by allowing the cell membrane to undergo arrangements in its composition to be able to cope with the pollutants in its environment. For instance, the lipid composition can be changed through alterations in the polar head groups or acyl chains of membrane glycerophospholipids. Bacterial cytoplasmic membranes can compensate for altered growth conditions by a process known as homeoviscous adaptation which biochemically changes the membrane so it can remain in the fluid phase even as the environment changes [76]. Or in the case of folate and vitamin B6, they are important for the growth of pathogenic organisms. At conserved sites, the molecular functions involved in the synthesis of molecules with biotechnological applications, such as retinol metabolism, and sesquiterpenoid and triterpenoid biosynthesis were significantly represented. Retinol is used in cosmetic and dermatological treatment against anti-wrinkle treatments, improvement of skin texture, depigmentation, dryness, and expression lines; and the market size has been estimated to be about $1.6 billion worldwide. Its mechanism of action is through the inhibition of the expression of collagenase and matrix metalloproteinases (MMP); as well as the stimulation of the synthesis of type 1 collagen and glycosaminoglycans (GAGs) [77,78,79]. Today the bioproduction of retinoids is sought through the use of bacteria. Terpenoids, such as sesquiterpenoids and triterpenoids, due to their structural diversity have a wide range of industrial applications as pharmaceuticals, flavorings, fragrances, antimicrobials, pesticides, and antibiotics, among others [80,81]. Recent genome mining studies suggest that bacteria are a potentially much richer source of terpene synthases (TS), including enzymes from Cyanobacteria and Proteobacteria. Actually new terpenoid products have been detected in bacterial species, including aromandendrene, acora-3.7 (14) -diene, and longiborneol [80], opening the possibility of finding in bacteria a source of new terpenoids with potential biotechnological use. Finally, our results indicate that conserved wetlands represent an invaluable source of molecules with potential applications in different industries such as pharmaceutical, cosmetic, agricultural, among others. As it has been reported in the case of the Non-ribosomal peptides synthetase (NRPSs) genes, which synthesize compounds with different biotechnological applications and whose presence is enriched in El Palmar [82]. It is important to mention that metabolic functions associated with biogeochemical processes and degradation of xenobiotics were also identified. However, its relative abundance did not present statistical significance in any of the locations analyzed. In general, biogeochemical processes show a similar relative abundance at all sampled locations. Which leads us to interpret that the biogeochemical processes carried out by prokaryotic organisms remain homogeneous along the Yucatan coast. Regarding the degradation of xenobiotics, their presence in the locations analyzed in our study shows a differential presence. Whereas in the 2019 samples, there is a predominance of aromatic compounds, in the 2017 and 2018 samples, there is a predominance of alkane derivatives. It is important to note that aromatic compounds can be important growth substrates for microorganisms; and in the case of toluene it serves as sources of carbon and energy for the growth of bacteria [65,83].

## 5. Conclusions

To our knowledge, this is the first study where the prokaryotic diversity of the Yucatan coastal wetland is described using massive sequencing tools, in order to know the changes in the microbial landscape. The study was carried out in four wetland sediments of Yucatan Peninsula: the first two are located in Sisal and Progreso, with a high impact by anthropogenic activities, whereas the other two belong to the ecological reserve El Palmar and Bocas de Dzilam, with almost nothing anthropogenic activities. Our analysis revealed changes in the composition and abundance of the prokaryotic communities of the Yucatan coast. For instance, the identification of ecologically important bacterial species from Planctomycetes and Acidobacteria phyla suggests that Palmar and Bocas de Dzilam wetland sediments are well-preserved. On the contrary, the identification of candidate phylum FBP and Chloroflexi in sediments of Sisal and Progreso, suggests that both wetlands are contaminated. We consider that an exhaustive sampling, monitoring the locations along the year, is needed not to only validate our results and to identify biological indicators of coastal environmental states in the medium term, but also to propose protection mechanisms of the contaminated locations. Additional transcriptomic studies must be conducted to validate the metabolic functions predicted in this study, and to allow us to identify molecular biomarkers for the development of molecular tools for environmental monitoring. As well as for the evaluation of the biotechnological potential through the identification of bioactive compounds, such as retinol metabolism, and sesquiterpenoid and triterpenoid biosynthesis, of the coastal zone of Yucatan.

## Data Availability

Data available upon express request.

## Supplementary Materials

The following are available online at https://www.mdpi.com/article/10.3390/microorganisms9040877/s1, Supplementary Table S1. Statistical data of the paired end reads for each sample analyzed in this study. Supplementary Table S2. Reads assigned to bacterial OTUs. Supplementary Table S3. Estimation of alpha diversity of sampling sites of wetlands from Yucatan. Supplementary Table S4. Metabolic functions identified. Supplementary Figure S1. Sampled sites at Sisal and Progreso. Supplementary Figure S2. Relative abundance of bacteria composition and their standard deviation across the four wetland sampling sites. Supplementary Figure S3. Rarefaction curves-based of the phylogenetic prokaryotic diversity of the studied sites. Supplementary Figure S4. Metataxonomic profile comparisons of differentially phylotypes between conserved sites (Bocas de Dzilam and El Palmar) and contaminated sites (Progreso and Sisal) using STAMP analysis. Supplementary Figure S5. Metabolic pathways profile comparisons between study sites.
