# Supplementary material for: Comparing Sediment Microbiomes in Contaminated and Pristine Wetlands along the Coast of Yucatan
Source: Microorganisms. 2021 Apr 20;9(4):877. doi: 10.3390/microorganisms9040877 (PMC8073884; doi:10.3390/microorganisms9040877)
Supplement: Supplementary file 1 [file microorganisms-09-00877-s001.zip › Supplementary_Material/Supplementary_Table_S1.pdf]

**Supplementary Table S1.** Statistical data of the paired end reads for each sample analyzed in this study from four different locations. SD: Standard deviation; %: Percentage; F: forward; R: reverse.

| Location (year) | Number of Reads | Total bases | Mean read length (SD) | Number of Filtered Reads (%) |
|---|---|---|---|---|
| *Bocas de Dzilam* (2019) | 93,929 **F** | 28,154,347 | 299.7 (11.5) | 63,894 (68.02) |
| | 93,929 **R** | 28,090,873 | 299.1 (11.3) | |
| *Bocas de Dzilam* (2019) | 82,638 **F** | 24,767,807 | 299.7 (12.7) | 62,267 (75.35) |
| | 82,638 **R** | 24,698,252 | 298.9 (12.8) | |
| *Bocas de Dzilam* (2019) | 93,922 **F** | 28,115,237 | 299.3 (15) | 70,047 (74.58) |
| | 93,922 **R** | 28,030,213 | 298.4 (15) | |
| *El Palmar* (2018) | 56,963 **F** | 17,136,763 | 300.8 (3.1) | 23,974 (42.09) |
| | 56,963 **R** | 17,076,155 | 299.8 (7.3) | |
| *El Palmar* (2018) | 47,789 **F** | 14,375,024 | 300.8 (2) | 19,568 (40.95) |
| | 47,789 **R** | 14,329,472 | 299.8 (6.2) | |
| *El Palmar* (2017) | 39,937 **F** | 12,014,595 | 300.8 (3.2) | 28,409 (71.13) |
| | 39,937 **R** | 11,970,763 | 299.7 (7.4) | |
| *Progreso* (2019) | 102,655 **F** | 30,814,442 | 300.2 (10) | 78,072 (76.05) |
| | 102,655 **R** | 30,726,022 | 299.3 (10) | |
| *Progreso* (2019) | 72,939 **F** | 21,868,651 | 299.8 (12.1) | 43,510 (59.65) |
| | 72,939 **R** | 21,807,806 | 299.0 (12) | |
| *Progreso* (2019) | 89,402 **F** | 26,728,096 | 299 (16.1) | 64,953 (72.65) |
| | 89,402 **R** | 26,650,848 | 298.1 (16.1) | |
| *Sisal* (2018) | 45,020 **F** | 13,548,414 | 300.9 (2.5) | 21,347 (47.42) |
| | 45,020 **R** | 13,496,115 | 299.8 (7.2) | |
| *Sisal* (2018) | 44,925 **F** | 13,512,248 | 300.8 (3.6) | 16,896 (37.61) |
| | 44,925 **R** | 13,468,107 | 299.8 (7.1) | |
| *Sisal* (2017) | 38,421 **F** | 11,553,496 | 300.7 (4.6) | 27,020 (70.33) |
| | 38,421 **R** | 11,514,926 | 299.7 (8.2) | |
