# Supplementary material for: Comparing Sediment Microbiomes in Contaminated and Pristine Wetlands along the Coast of Yucatan
Source: Microorganisms. 2021 Apr 20;9(4):877. doi: 10.3390/microorganisms9040877 (PMC8073884; doi:10.3390/microorganisms9040877)
Supplement: Supplementary file 1 [file microorganisms-09-00877-s001.zip › Supplementary_Material/Supplementary_Table_S3.pdf]

Supplementary Table S3. Estimation of alpha diversity of sampling locations of wetlands from Yucatán. %: Percentage; SE: Standard error.

Sample (year)	Observed	Shannon index	Chao1 (SE)
Bocas de Dzilam (2019)	384	5.53	384 (0.02)
Bocas de Dzilam (2019)	466	5.89	466 (0.08)
Bocas de Dzilam (2019)	420	5.73	420 (0)
El Palmar (2018)	179	4.91	179 (0)
El Palmar (2018)	147	4.68	147 (0)
El Palmar (2017)	191	5.0	191 (0)
Progreso (2019)	282	5.22	282 (0)
Progreso (2019)	188	4.79	188 (0.27)
Progreso (2019)	271	5.09	271 (0.01)
Sisal (2018)	49	3.34	49 (0)
Sisal (2018)	117	4.47	117 (0)
Sisal (2017)	204	5.06	204 (0)
