# Supplementary material for: Comparing Sediment Microbiomes in Contaminated and Pristine Wetlands along the Coast of Yucatan
Source: Microorganisms. 2021 Apr 20;9(4):877. doi: 10.3390/microorganisms9040877 (PMC8073884; doi:10.3390/microorganisms9040877)
Supplement: Supplementary file 1 [file microorganisms-09-00877-s001.zip › Supplementary_Material/Supplementary_Figures.pdf]

Microbiome landscape changes of the coastal of Yucatán

Herón Navarrete-Euan[1], Zuemy Rodriguez-Escamilla[1], Ernesto Pérez-Rueda[2], Karla Escalante-Herrera[1] and Mario Alberto Martinez-Nuñez[1 *,]*

[1] UMDI-Sisal, Facultad de Ciencias, Universidad Nacional Autónoma de México. Mérida, Yucatán, México; heronnavarrete@hotmail.com (H.N.E.); zuemy.rodriguez@gmail.com (Z.R-E.); susana_escalanteh@hotmail.com (K.E-H.)
[2] Instituto de Investigaciones en Matemáticas Aplicadas y en Sistemas, UNAM. Unidad Académica Yucatán. Mérida, Yucatán, México; ernesto.perez@iimas.unam.mx
* Correspondence: mamn@ciencias.unam.mx; Tel.: +52 999 3410860, ext. 7631.

Supplementary Figures.

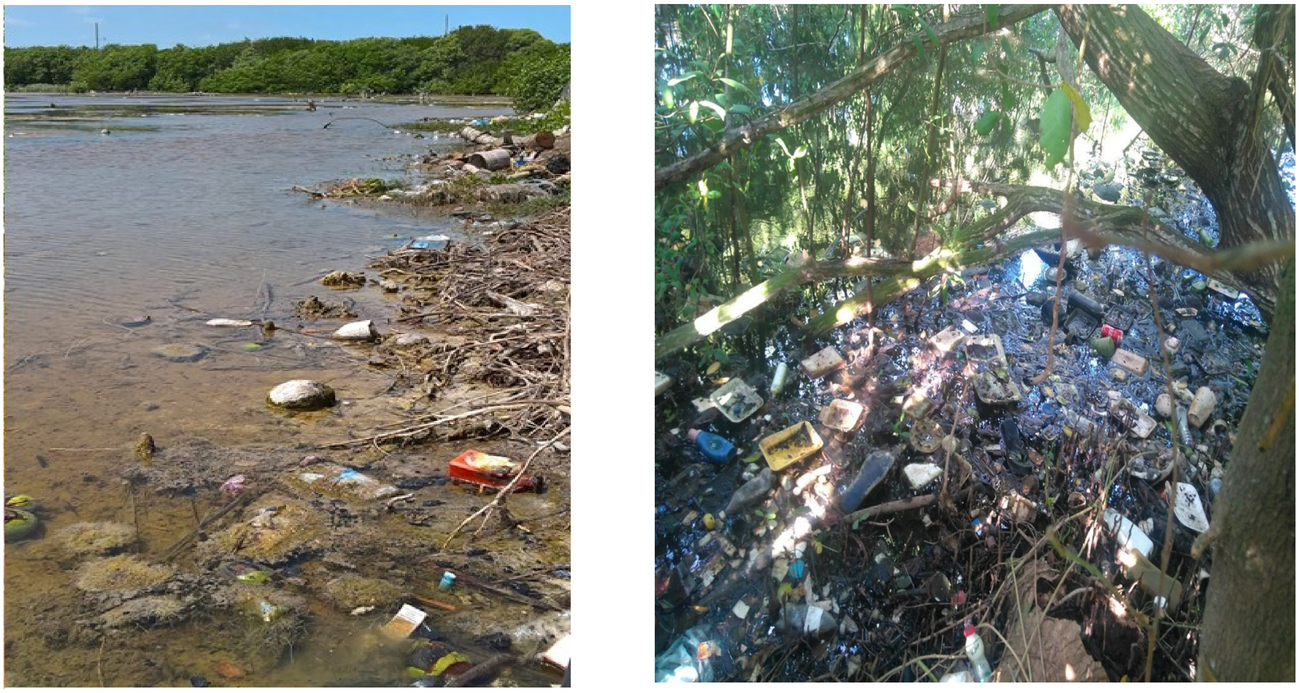

Supplementary Figure S1. Sampled sites at Sisal (left) and Progreso (right). The garbage deposited in the wetlands by the residents can be observed.

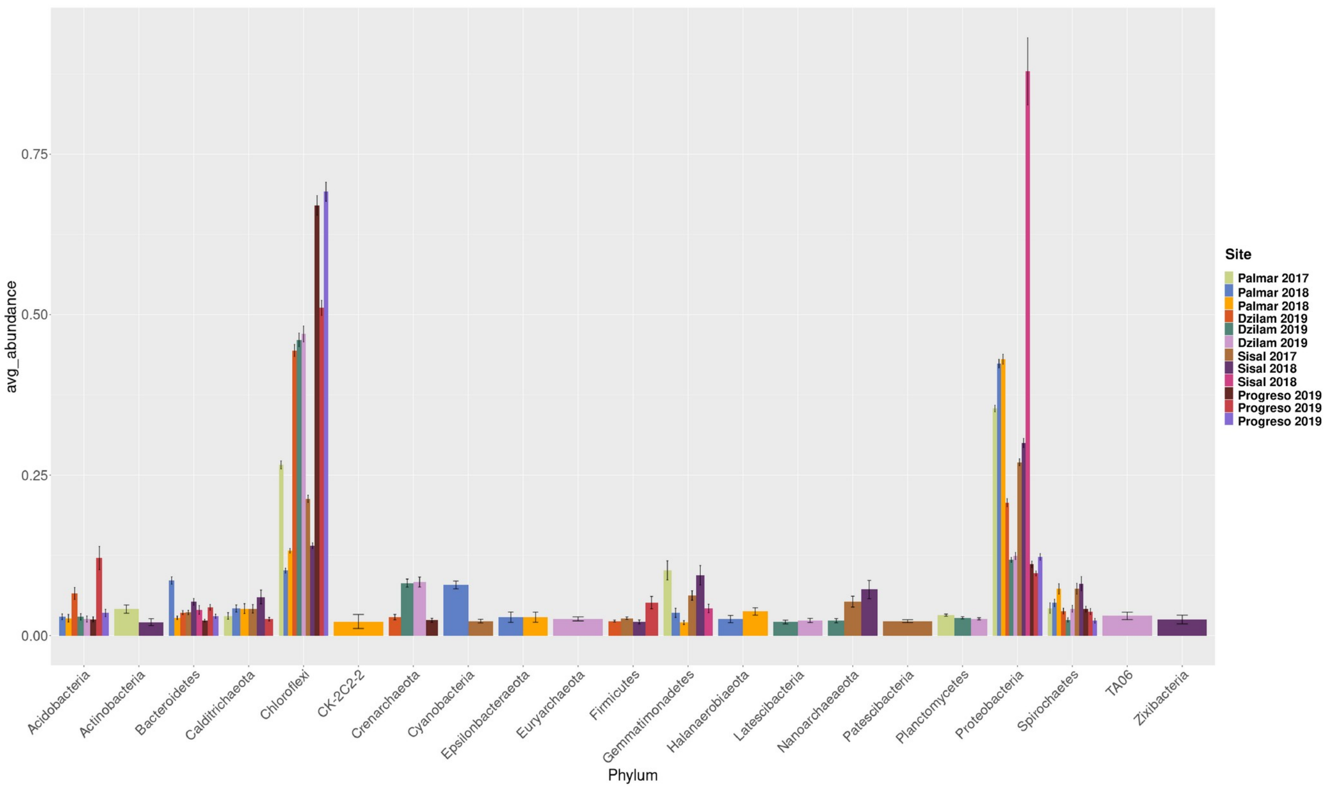

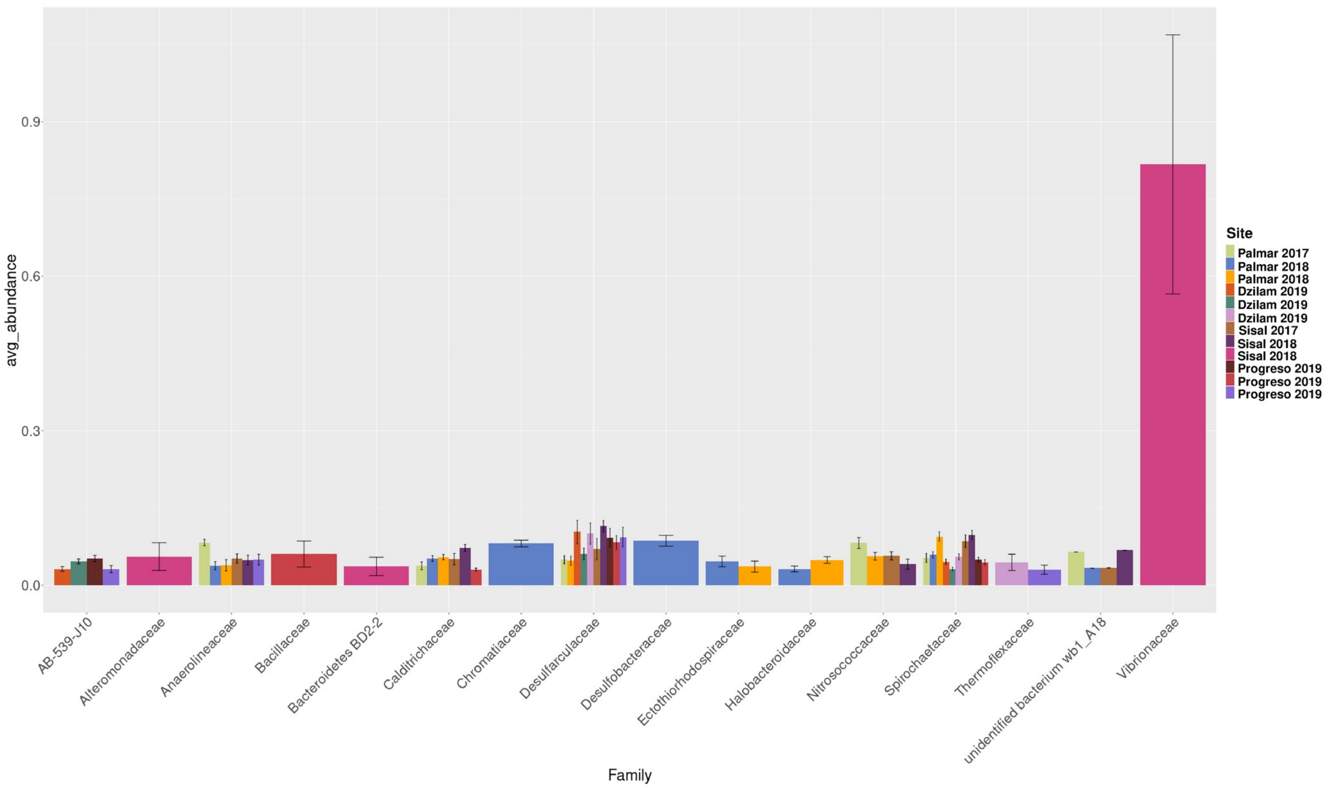

Supplementary Figure S2. Relative abundance of bacteria composition and their standard deviation across the four wetland sampling sites at (upper) phylum and (bottom) family levels. Bocas de Dzilam: conserved site; El Palmar: conserved site; Progreso: contaminated site; Sisal: contaminated site. X-axis: taxon. Y-axis: relative abundance of taxon.

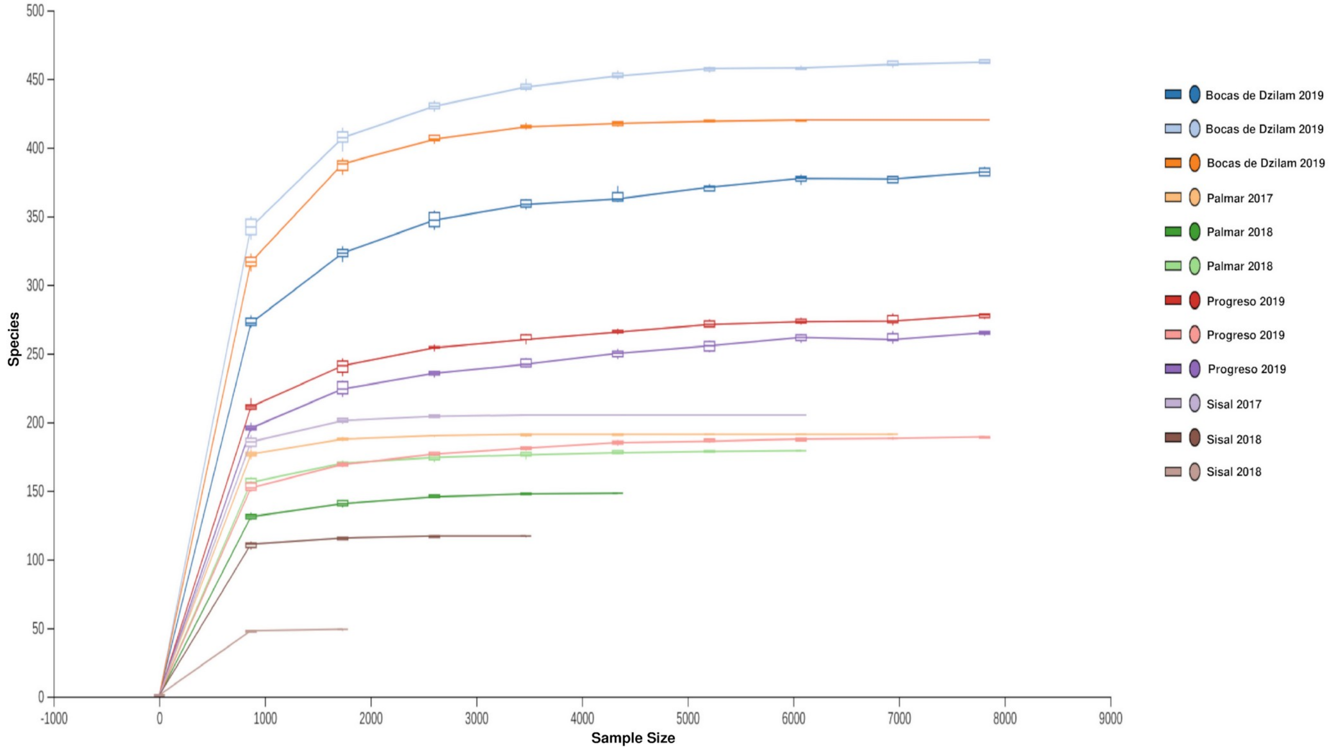

Supplementary Figure S3. Rarefaction curves-based of the phylogenetic prokaryotic diversity of the studied sites. X-axis: Number of species annotated. Y-axis: Sample size as number of reads obtained from sequencing.

A

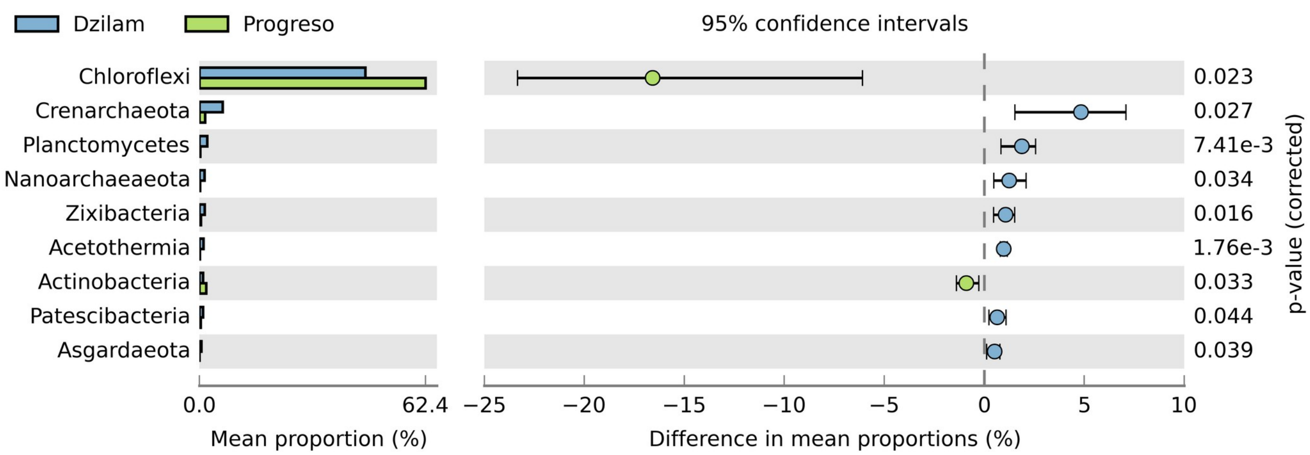

B

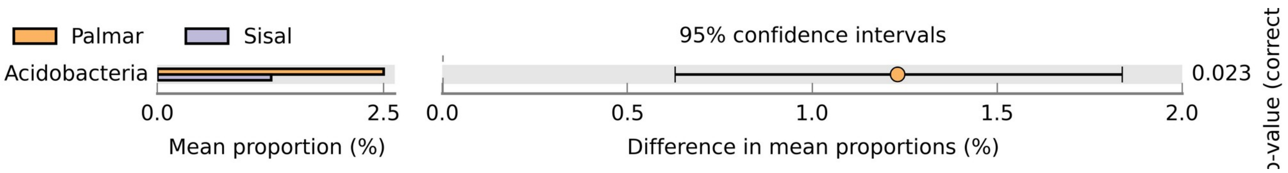

Supplementary Figure S4. Metataxonomic profile comparisons of differentially phylotypes between conserved sites (Bocas de Dzilam and El Palmar) and contaminated sites (Progreso and Sisal) using STAMP analysis. **A**: analysis at phylum level of Bocas de Dzilam and Progreso samples. **B**: analysis at phylum level of Palmar and Sisal samples.

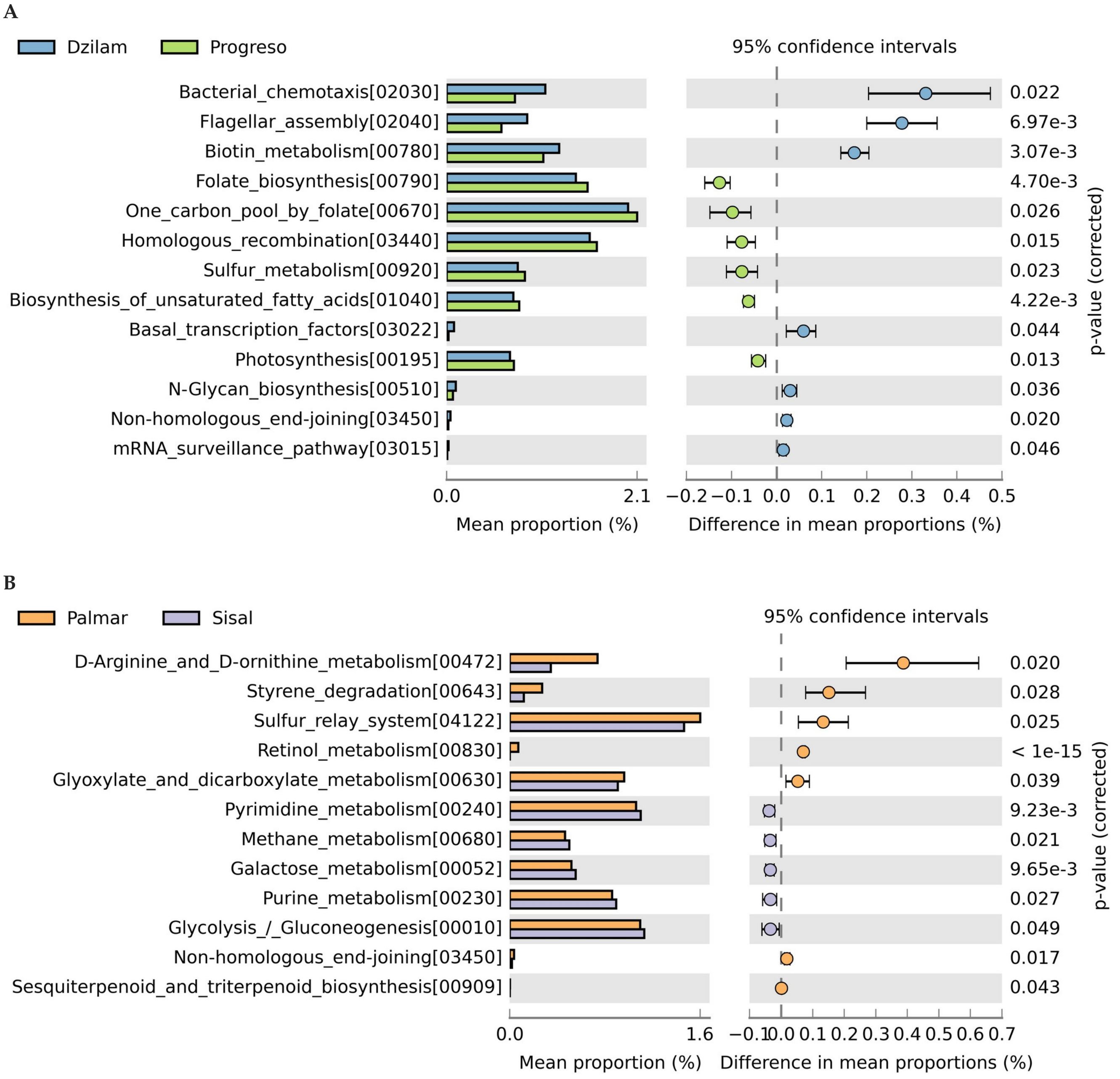

Supplementary Figure S5. Metabolic pathways profile comparisons between study sites. **A:** Bocas de Dzilam and Progreso. Blue bar: conserved site; green bar: contaminated site. **B:** El Palmar and Sisal. Orange bar: conserved site; purple bar: contaminated site. Statistical analyzes were done with STAMP analysis. Analyses were carried out at level 3 of specific pathway associated with a specific function using KEGG database.
